# Applying WCACG modified process is beneficial on reduced door-to-balloon time of acute STEMI patients

**DOI:** 10.1051/bmdcn/2019090210

**Published:** 2019-05-24

**Authors:** Qiao-Wen Li, Xiao-Jian Liu, Jin-Hua Li, Guo-Qi Zhang, Su-Min Chen, Chao-Long Huang, Min Qiu, Yue-Liang Li, Peng Duan, Yi-Jiun Weng, Xiao-Yong Zhang, Chih-Yang Huang

**Affiliations:** 1 Department of Cardiology, Qingyuan People’s Hospital, The Sixth Affiliated Hospital of Guangzhou Medical University Guangdong China; 2 Graduate Institute of Basic Medical Science, China Medical University Taichung 404 Taiwan; 3 Graduate Institute of Chinese Medical Science, China Medical University Taichung 404 Taiwan; 4 Department of Health and Nutrition Biotechnology, Asia University Taichung 413 Taiwan

**Keywords:** Door-to-balloon time, ST-elevation myocardial infarction, WeChat application communication group (WCACG)

## Abstract

Background: Various systems have employed with the objective to reduce the time from emergency medical services contact to balloon inflammation for ST-elevation myocardial infraction (STEMI) patients. The WCACG message system was used to an alternative communication platform to improve confirmation of the diagnosis and movement to treatment, resulted in shorten the door-to-balloon (D-to-B) time for STEMI patients.

Methods: We collected 366 STEMI patients admitted at the Sixth Affiliated Hospital of Guangzhou Medical University, Qingyuan People’s Hospital, Department of Cardiology, during the period from June 2013 to October 2015. The patients were divided into two groups one underwent the current GC processes and the other group was handled using WCACG system. We compared between two groups with several indicators including D-to-B time, duration of hospitalization, associated costs, and incidence of adverse cardiovascular events.

Results: The results show that the new method with WCACG system significantly reduced the average D-to-B time (from 100.42 ± 25.14 mins to 79.81 ± 20.51 mins, *P* < 0.05) compared to the GC processes, and also reduced the duration, costs and undesirable cardiac incidence during hospitalization.

Conclusions: The modified WCACG process is an applicable system to save pieces of time and efficiently integrate the opinions of experts in emergency.

## Introduction

AMI, Acute myocardial infarction; CABG, Coronary Artery Bypass Grafting; D-to-B, door-to-balloon; NSTEMI, non-ST segment elevation acute myocardial infarction; PCI, percutaneous coronary intervention; STEMI, ST-elevation myocardial infarction; TIMI, thrombolysis in myocardial infarction; WCACG, WeChat application communication group; GC, Green Channel

Acute myocardial infarction (AMI) is a serious and common coronary artery disease [1, 2]. The occurrence of AMI in coronary blood supply will be drastically reduced or interrupted where corresponding myocardial ischemic necrosis caused by severe and persistent acute ischemia has occurred [3]. The AMI includes acute ST segment elevation myocardial infarction (STEMI) and non-ST segment elevation acute myocardial infarction (NSTEMI) [4]. Timely reperfusion therapy can reduce heart failure risk, reduce infarct size, achieve and maintain normal blood perfusion in myocardial tissue[5, 6]; it can also prevent infarct expansion and inhibit left-ventricular remodeling of STEMI that has happened within 12 hrs to continuous ST-segment elevation and also in new-onset left bundle branch block patients [7, 8]. Thus, percutaneous coronary intervention (PCI) has become a major and most effective reperfusion therapy [9–11].

With the increasing popularity of interventional treatment technology, more number of hospitals has implemented the emergency PCI [12–14]. However, mortality has not significantly decreased in STEMI patients and has also missed optimum reperfusion time due to delay in treatment after the onset of STEMI and medical diagnosis processes [15–17]. Some reports have already proved that the timely application of reperfusion surgery can bring in more benefits to STEMI patients [18–23]. The prognosis of STEMI patients after the first PCI had a direct relationship with door-to-balloon (D-to-B) time. Thus, the D-to-B time reduction and ratio of increase in early/delayed reperfusion are the most necessary requirements for STEMI patient treatments [24–26]. Time delay within the health care system is the most serious problem that has to be solved on a first priority basis.

**Fig. 1:**
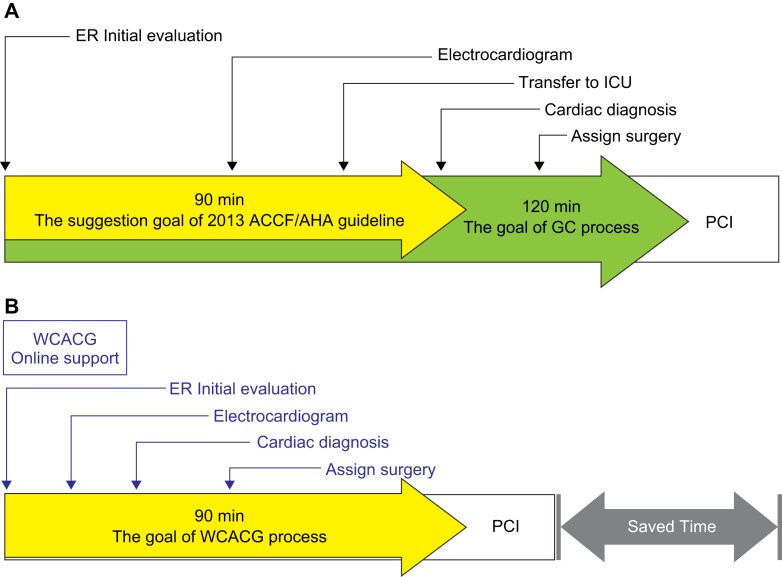
D-to-B time cost of GC modified process and WCACG modified process. (A) GC process reduced D-to-B time process cost by 100 min from over 4 h originally traditional diagnosis process, but still wastes a lot of time on cardiac catheterization and diagnosis after the STEMI patient’s arrival at the emergency department. (B) Based on green channel modified process, WeChat modified process is a major help in reducing the time spent on cardiac catheterization, diagnosis, dissections between doctors and surgery preparation.

In 2010, a new process named the Green Channel (GC) modified process was applied and replaced the traditional medical diagnosis processes in the Sixth Affiliated Hospital of Guangzhou Medical University, Qingyuan People’s Hospital. In average, GC modified process applied to STEMI patients will greatly extend the D-to-B time, reduce STEMI treatment success rate, mortality and length of stay. In brief, the GC process helps STEMI patients in the reduction of D-to-B time by 120 min from the previous figure that exceeded 4 h in average during 2010 to 2014. (Figure 1A) Both U.S. AHA/ACC and China STEMI diagnosis suggested the time required within 90 min from arrival at hospital to first balloon dilatation in STEMI patients as a treatment guideline [27]. In this study, average D-to-B time, average of hospitalized days and hospitalization costs, impact of adverse cardiovascular events during hospitalization and the incidence of other indicators of STEMI patients are evaluated by using a modern communications technology through the establishment of specialist consultations, WCACG and simplified AMI treatment processes (Figure 1B). Based on the outcome of this evaluation, a better version of STEMI medical diagnosis process can be developed.

## Materials and methods

### Setting of We-Chat application contact group (WCACG)

On August 1, 2014, WCACG was established as the base in GC process. The WCACG consisted of cardiovascular medicine specialist and emergency department physician, cardiology specialists and doctors. In emergencies, department doctors first diagnosed eighteen-lead ECG and uploaded pictures of WCACG and then telephonically notified on-duty cardiology experts of judgments based on the patient’s chest pain and ECG time performance for assessment; these included the indications for surgery, surgical difficulty, risk, prognosis, preoperative intraoperative need of assistance and other factors (such as temporary pacemaker, breathing, etc.). After deciding on surgery, cardiology experts notified surgery personnel for improving preoperative preparation. This program was approved by Ethical Committee of Qingyuan People’s Hospital, Guangdong, China.

### Exclusion criteria of patients

This investigation comprised 366 patients with STEMI first observed from 2013 to 2015. All the objects were confirmed by medical history, physical examination, laboratory tests and laboratory examinations; these excluded Coronary Artery Bypass Grafting (CABG) patients with a history of coronary heart disease, severe systemic disease, inflammatory inhibitors, merger myocarditis, severe cardiac dysfunction, peripheral vascular disease, stroke, severe liver and kidney diseases, autoimmune diseases and blood diseases. Furthermore, the need for coronary artery bypass grafting as exclusion criteria was studied. Accordingly, all patients were briefed on consent procedures that were compliant with modern standards of medical ethics.

### Groups design

All STEMI patients involved were divided into WGACG groups and the GC group. Two groups were formed according to gender, smoking, family status, average age, diabetes, hypertension, hyperlipidemia, major vascular stenosis and history of premature coronary heart disease (Family history of premature coronary heart disease). If there had been angina comparative history, past history of PCI, stent placement number, etc., the difference was not statistically significant. The comparable (Table 1) criterion for smokers was as below: Current smokers (≥ 5 cigarettes/day) or having smoking history of more than 10 years but quitting smoking only for less than a year. According to the Chinese dyslipidemia prevention advice of 2006, hyperlipidemia diagnostic criteria were defined thus “Hypercholesterolemia (total cholesterol ≥ 6.22 mmol/L and/or low-density lipoprotein cholesterol ≥ 4.14 mmol/L), hypertriglyceridemia (triglycerides ≥ 2.26 mmol/L) and combined hyperlipidaemia”. The above-mentioned information, age and the number of implantations were processed as measurement data while all the others were processed as count data.

**Table 1:** Comparison between GC modified process and WCACG modified process for STEMI patients.

Characteristic		WCACG (n = 195)	GC (n = 171)	x2/t	*P* value
Gender (male), n(%)		149(76.4%)	123(71.9%)	x2 = 0.958	0.340
Age (mean ± SD, years)		60.45 ± 12.61	62.29 ± 12.44	t = 1.406	0.778
Smoking, n(%)		71(36.4%)	77(45.0%)	x2 = 2.810	0.109
live alone		13(6.7%)	5(2.9%)	x2 = 2.729	0.145
Family history of early CAD, n(%)		11(5.6%)	11(6.4%)	x2 = 0.101	0.827
Angina pectoris (n%)		26(13.3%)	30(17.5%)	x2 = 1.246	0.309
History of diabetes, n(%)		52(26.7%)	48(28.1%)	x2 = 0.09	0.814
History of hypertension, n(%)		74(37.9%)	67(39.2%)	x2 = 0.220	0.669
History of hyperlipidemia, n(%)		66(33.8%)	62(36.3%)	x2 = 0.233	0.661
History of PCI, n(%)		3(1.5%)	5(2.9%)	x2 = 0.092	1
lesion number	1 branch	68	73		
	2 branches	51	45	x2 = 3.093	0.213
	≥3 branches	76	53		
Killip level	I	187(95.9%)	165(96.5%)
	II	8(4.1%)	6(3.5%)	x2 = 0.087	0.793
Number of implantations (mean ± SD)		1.16 ± 0.385	1.15 ± 0.376	t = 0.302	0.562

### Treatments

Major vascular stenosis ≥ 70% of patients with STEMI, are to be PCI; all patients included in this study underwent stenting. STEMI patients without contraindications LMWH 3-5 days are longterm aspirin, clopidogrel, statins, β-blockers and / or of ACEI and / or ARB of drugs. Smoker patients have to quit smoking and hypoglycemic therapy needs to be administered to patients with diabetes and high blood pressure patients. The related indicators then have to be monitored.

### Statistical methods

The Statistical analysis in this work using SPSS 16.0 statistical software or data was presented as Mean ± SD. Two groups were compared using *T* test measurement data differences using chisquare test of difference between the two groups. Count data and Pearson correlation analysis between two variables along with *P* < 0.05 were statistically significant.

## Results

### WGACG *vs.* GC group

The WCACG modified process can reduce the D-to-B time from 100 mins to 80 mins compared with GC group. The length of stay along with hospital charges was lower than the GC group (*P* < 0.05, Table 2). Hospital costs correlation analysis in patients with STEMI D-to-B time, hospital stay and hospital costs were positively correlated (r = 0.357, *P* < 0.001; r = 0.327, *P* < 0.001).

**Table 2:** GC modified process and WCACG modified process on average, D-to-B time, average hospitalization expenses and the change in length of hospital stay.

Process		n	D-to-B time (min)	hospitalized costs (104RMB)	hospital length of stay (day)
WCACG		195	79.81 ± 20.51	5.17 ± 1.87	7.30 ± 2.76
GC		171	100.42 ± 25.14	5.69 ± 2.31	8.49 ± 3.23
	*t*		8.632	2.373	3.792
	*P* value		0.026	0.002	0.029

### WGACG group and the GC group after the culprit artery

The incidence of adverse cardiovascular events during hospitalization, thrombolysis in myocardial infarction (TIMI) grade and the WGACG process were compared with the GC process of patients. The culprit artery blood flow (Table 3) TIMI grade after no significant difference (*P* > 0.05) showed that the adverse car diovascular events during hospitalization were significantly lower than the GC group (*P* < 0.05).

**Table 3:** Comparison between GC modified process and WCACG modified process for postoperative TIMI flow grade and incidence of adverse cardiovascular events during the period of hospitalization.

Characteristic		WCACG (n = 195)	GC (n = 171)	x2/t	*P* value
TIMI flow grade	II	11	6
	III	184	165	x2 = 0.935	0.457
Incidence of adverse cardio-vascular events during the periodof hospitalization, n(%)		6(3.18%)	15(8.77%)	x2 = 5.283	0.025

## Discussions

The distance traversed by the STEMI patient’s journey to the hospital was random and unpredictable; this droved to minimize the working hours available after the hospital admission process of the patients. Cardiac characterizations and diagnosis also needed a time slot for reducing the man-made waiting time and speeding up the communication between doctors [28, 29]. Wide usage of smartphones has enabled novel opportunities to improve clinical practices by integrating to mobile technologies [30–32]. WCACG usage of network communications software can then receive the patient’s calls when they start so that the attending physician for patients before the hospital admission can obtain the patient’s condition and arrange inspection procedures; this would ensure that the waiting time for the patient at the hospital after the checkin is reduced [33].

The results can also be uploaded to the WCACG system, allowing multiple numbers of professional physicians to discuss, diagnose, and arrange for the necessary surgical procedures. The results of this study showed that the D-to-B time of the new mode group, the length of hospital stay and hospital charges were significantly lower than the GC mode group. By using WCACG mode, the STEMI patient’s treatment process was simplified and consequently, the average D-to-B time was reduced from 100 min (GC group) to 80 min thereby reaching the 2013 American College of Cardiology Foundation (ACCF) / American Heart Association (AHA) STEMI patient’s management requirements [34].

The results suggest that the new STEMI treatment process developed in this study can improve the efficiency of treatment in patients with STEMI when compared with previous patient visits. This has greatly reduced the patient’s average D-to-B time. In addition, the study also showed that patients’ D-to-B time, hospital stay and hospital costs were positively correlated. By shortening STEMI patients’ D-to-B time, myocardial ischemia time can be reduced; this also decreases the scope of myocardial infarction [35]. The incidence of complications is thus brought down and alleviates the patient’s symptoms quickly; bodily functions can also recover faster [36, 37]. Hence hospital stay is shortened, hospital costs are reduced and this optimizes the usage of valuable medical resources.

This work has demonstrated clearly that after the occurrence of AMI, sustained opening of infarct-related artery can be reduced in size to improve heart function and reduce mortality; this would improve the prognosis of patients with AMI [38]. The results of the study found that treatments using WGACG process simplified STEMI patients’ D-to-B time, reduced the incidence of adverse cardiovascular events during hospitalization and improved patient outcomes. This means that the new hospital STEMI patient treatment processes for improving prognosis of patients cannot be neglected. The study also found that the usage of WCACG improved the STEMI patient’s treatment after the occurrence of vascular blood flow which was consistent with the measures outlined by de Waard GA *et al*. and Pan W *et al*. [39, 40]. Thus, TIMI grade had little impact, which again may be related to factors such as culprit artery blood flow, the relative lack of sample size and other factors (Table 3).

Currently, the establishment of regional co-treatment system in order to achieve the ultimate goal of chest pain center construction has become a hot spot in the field of STEMI patient treatment. The important goal is to shorten the STEMI patient’s D-to-B time and improve patient outcomes. The STEMI patient’s hospital treatment process using WCACG has not only reduced the D-to-B time but also improved patient outcomes, shortened hospital stay and reduced hospital costs. The WCACG in most domestic and foreign areas are feasible because the communication app is gaining popularity.
